# Semantic dementia Brazilian study of nineteen cases

**DOI:** 10.1590/S1980-57642008DN10400007

**Published:** 2007

**Authors:** Mirna Lie Hosogi Senaha, Paulo Caramelli, Claudia Sellitto Porto, Ricardo Nitrini

**Affiliations:** 1 Speech Pathologist, Member of Behavioral and Cognitive Neurology Unit of the Department of Neurology, University of São Paulo School of Medicine, São Paulo, Brazil.; 2 Associate Professor, Department of Internal Medicine, Faculty of Medicine, Federal University of Minas Gerais, Belo Horizonte, Minas Gerais, Brazil.; 3 Neuropsychologist, Member of Behavioral and Cognitive Neurology Unit of the Department of Neurology, University of São Paulo School of Medicine, São Paulo, Brazil.; 4 Behavioral and Cognitive Neurology Unit of the Department of Neurology, and Cognitive Disorders Reference Center (CEREDIC). Hospital das Clínicas of the University of São Paulo School of Medicine, São Paulo, Brazil.

**Keywords:** semantic dementia, semantic memory, fluent progressive aphasia, primary progressive aphasia, word comprehension, temporal lobe, demência semântica, memória semântica, afasia progressiva fluente, afasia progressiva primária, compreensão de palavras, lobo temporal

## Abstract

The term semantic dementia was devised by Snowden et al. in 1989 and nowadays, the semantic dementia syndrome is recognized as one of the clinical forms of frontotemporal lobar degeneration (FTLD) and is characterized by a language semantic disturbance associated to non-verbal semantic memory impairment.

**Objectives:**

The aim of this study was to describe a Brazilian sample of 19 semantic dementia cases, emphasizing the clinical characteristics important for differential diagnosis of this syndrome.

**Methods:**

Nineteen cases with semantic dementia were evaluated between 1999 and 2007. All patients were submitted to neurological evaluation, neuroimaging exams and cognitive, language and semantic memory evaluation.

**Results:**

All patients presented fluent spontaneous speech, preservation of syntactic and phonological aspects of the language, word-finding difficulty, semantic paraphasias, word comprehension impairment, low performance in visual confrontation naming tasks, impairment on tests of non-verbal semantic memory and preservation of autobiographical memory and visuospatial skills. Regarding radiological investigations, temporal lobe atrophy and/or hypoperfusion were found in all patients.

**Conclusions:**

The cognitive, linguistic and of neuroimaging data in our case series corroborate other studies showing that semantic dementia constitutes a syndrome with well defined clinical characteristics associated to temporal lobe atrophy.

## Abstract (pt)

O termo demência semântica foi lançado por Snowden et al. em 1989 e, atualmente, a síndrome da demência semântica é reconhecida como uma das formas clínicas da degeneração lobar fronto-temporal (DLFT) e é caracterizada por distúrbio semântico da linguagem associado a comprometimento semântico não-verbal.

**Objetivos:**

Este trabalho teve como objetivo descrever uma amostra brasileira de 19 casos de demência semântica, ressaltando as características clínicas importantes para a realização do diagnóstico diferencial desta síndrome.

**Métodos:**

Foram estudados 19 casos com demência semântica avaliados entre 1999 e 2007. Todos os pacientes foram submetidos à avaliação neurológica, exames de neuroimagem e avaliação cognitiva, da linguagem e da memória semântica.

**Resultados:**

Todos os pacientes apresentaram produção espontânea fluente, preservação dos aspectos sintáticos e fonológicos da linguagem, dificuldade em encontrar palavras, parafasias semânticas, dificuldade de compreensão de palavras, baixo desempenho em provas de nomeação por confrontação visual, falhas em provas que avaliam a memória semântica não-verbal e preservação da memória autobiográfica e de habilidades visuoespaciais. Em relação aos achados de neuroimagem, o comprometimento do lobo temporal (atrofia e/ou hipoperfusão) foi encontrado em todos os pacientes.

**Conclusões:**

Os achados cognitivos, lingüísticos e de neuroimagem do nosso grupo de pacientes corroboram outros estudos que mostram que a demência semântica constitui uma síndrome com características clínicas bem definidas associada à atrofia do lobo temporal.

Currently, it is acknowledged that a language disturbance can often be the first symptom of a neurodegenerative disease and remain the main manifestation of the disease for a significant period. The presence of a progressive and predominant language disturbance in the first two years, this being the only factor compromising one of the main criteria for the diagnosis of the primary activities of daily living is progressive aphasia syndrome (PPA).^1-4^ The existence of this syndrome was reported by Mesulam^5^ in 1982 through the publication of a paper in which five cases were described with progressive language deterioration without a generalized dementia, associated to left perisylvian region atrophy.

After the publication of this seminal article by Mesulam^5^ there was great interest in studies on PPA and within a decade more than 100 cases had been reported in the literature.^3,6-7^ Heterogeneity of the clinical forms of this syndrome became apparent from the different publications. Typically, the PPA initial phase is characterized by an anomic stage. With disease progression, different manifestations can be observed: the anomia continues as a universal finding, but disturbances in the semantic, phonological and syntactic aspects of oral and written language vary considerably. Thus, the fluency criteria (articulation, flow, and number of words per utterance) began to be used for the division of the PPA forms. Therefore, the PPA, after the initial anomic stage, has been classified into: non-fluent progressive aphasia (NFPA) with and without agrammatisms, and fluent progressive aphasia (fPPA) with comprehension deficits.

According to Mesulam,^2^ patients with NFPA with agrammatism present deficits in the syntactic and phonological aspects of the language in a very similar way to the classic Broca’s aphasia. The main characteristics found are: word-finding deficits, fluency impairment, production of short sentences (telegraphic) with tendency toward grammatical word absence and difficulties in complex sentences comprehension. The NFPA without agrammatism, also called logopenic progressive aphasia (LPA), occurs from the intensification of the anomia which started in the anomic stage, in such a way that the patient’s speech is marked by long pauses. In addition, LPA patients present production of correct syntactically simple sentences, along with difficulties in syntactic comprehension and preservation of semantic comprehension. The fPPA with comprehension deficits is characterized by spontaneous speech fluency, anomia and word comprehension impairment.

The term semantic dementia (SD) was devised in 1989 by Snowden et al.^8^ through the publication in which three patients who presented progressive semantic impairment, characterized by deficits in naming and comprehension of words and objects were described. The patients presented fluent speech, anomia and difficulties in understanding the meaning of words, in spite of the preservation of sentence comprehension. Associated to the language semantic disturbance, the patients presented difficulties in recognizing and identifying objects despite the preservation of perceptual abilities.

Another important study on SD characterization was published by the Cambridge group in 1992. Hodges et al.,^9^ from a description of five cases, proposed that SD course incorporated the following characteristics:

(1) selective impairment of semantic memory causing severe anomia, spoken and written single-word comprehension impairment, reduced generation of exemplars on category fluency tests and an impoverished general knowledge;
(2) relative sparing of syntax and phonology;
(3) normal perceptual skills and non-verbal problem-solving abilities;
(4) relatively preserved autobiographical and episodic memory;
(5) surface dyslexia.

The researchers mentioned that some cases with compatible characteristics with SD had been described in the literature under the PPA nomenclature because the main manifestations of SD are language problems. For this reason, they suggest that the use of the SD label should be more pertinent for the cases of fPPA related to verbal and nonverbal semantic knowledge impairment, because the alterations of these patients are not limited to linguistic aspects. Hodges et al. also proposed that the PPA term should be used only for NFPA patients, who have language verbal output deficits with preservation of word comprehension and nonverbal semantic knowledge. From this publication by the Cambridge group, the SD terminology began to be used by some researchers, as synonymous to fPPA and controversies in the literature and in clinical practice regarding the differentiation between PPA and SD started emerged. Some researchers use the SD label to designate the fluent subtype of PPA, while according to others, SD constitutes a new syndrome.

Consequently, some groups started attributing PPA only to those patients with NFPA and the SD term to patients with fPPA. This classification, contrasting NFPA and SD, was used in the consensus on clinical diagnostic criteria of frontotemporal lobar degeneration (FTLD)^10^. However, this idea is not unanimous: for some researchers, including Mesulam, the notion that PPA is always non-fluent is incorrect.^1-2,11^ These researchers defend the idea that PPA includes cases of non-fluent and fluent progressive aphasias.

The consensus on clinical diagnostic criteria of FTLD^10^ published in 1998, established the characteristics of the three main prototypal clinical different syndromes of FTLD: frontotemporal dementia, NFPA and SD. In this consensus, the core features of the NFPA are:

(a) insidious onset with gradual progression and
(b) nonfluent spontaneous speech with at least one of the following: agrammatism, phonemic paraphasias, and anomia.

Also in the consensus, SD is characterized as “semantic aphasia and associative agnosia” and the core features established are:

(a) insidious onset and gradual progression,
(b) fluency, empty spontaneous speech,
(c) loss of word meaning,
(d) semantic paraphasias and/or prosopoagnosia and/or,
(f) associative agnosia,
(g) preserved perceptual matching and drawing reproduction,
(h) preserved single-word repetition,
(i) preserved ability to read aloud and to write to dictation orthographically regular words.

Besides the controversies mentioned previously, another issue raised by the Cambridge group is related to the different interpretations given to the consensus on clinical diagnostic criteria of FTLD^10^ for SD, mainly over the issue regarding the presence of associative agnosia and/or prosopoagnosia associated to the aphasic disturbance.^12^ According to the Cambridge researchers,^12,13^ the designation of SD as “semantic aphasia and associative agnosia” creates confusion and divides opinion in the scientific field regarding the fPPA x SD question. However, for some researchers the diagnosis of SD is valid just for those patients whose gnosic impairment interferes in the activities of daily living, whereas for others such as the Cambridge group, the diagnosis of SD can be attributed to patients presenting mistakes in tests that evaluate nonverbal semantic knowledge in spite of the fact that this difficulty does not influence recognition of objects and family members in their daily life. Therefore, Adlam et al.^12^ suggest that the criteria of consensus for SD^10^ should be modified regarding the use of the agnosia term where this should be replaced by “compromise in tests of nonverbal associative knowledge.”

In relation to the anatomical aspects, Gorno-Tempini et al.^14^ carried out a study with 31 patients with PPA using voxel based morphometry on MRIs. Analyses of all patients showed that the left perisylvian region and the anterior temporal lobes were atrophied. In an analysis dividing the patients according to their clinical manifestations, the authors found the following data: NFPA with agrammatism was associated with left inferior frontal and insular atrophy;LPA was associated with left posterior temporal cortex and inferior parietal lobe, and SD was associated with anterior temporal involvement.

The objective of the present study was to describe a Brazilian sample of 19 cases of SD, emphasizing the clinical characteristics important for the differential diagnosis of this syndrome.

## Methods

Nineteen patients with SD were evaluated between 1999 and 2007. The concept of SD used in this study follows the definition proposed by Addlam et al.^12^

The patients were submitted to neurological examination, neuroimaging assessment and cognitive, language and semantic memory evaluation. All the patients were submitted to structural (MRI) neuroimaging assessment, except for one case that was submitted to structural (CT) assessment. Additionally, 13 patients were submitted to functional (SPECT) neuroimaging assessment. Most of the patients underwent full neuropsychological evaluation (14) although five patients were submitted to the brief cognitive evaluation.

All of the patients were submitted to the same language and semantic memory evaluation by the same researcher (MLHS) which included: communication functional evaluation, aphasia battery tests (Beta MT-86,^15^ Boston Diagnostic Aphasia Exam,^16^ Boston Naming Test,^17^ HFSP reading and writing protocols^18^) and tasks of semantic memory battery described in an earlier paper.^19^

## Results

Nineteen patients, 9 men and 10 women, were evaluated. The main demographic data and Mini-mental State Examination (MMSE)^20,21^ scores are shown in Table 1. All patients were right-handed aged between 58 and 88 years. Of the 19 patients, 11 (57.9%) had disease onset in the presenile phase.

**Table 1:** Demographic data of semantic dementia patients.

	Mean(SD*)	Minimum/ maximal values
Age	70.3 (9.2)	58-88
Age of onset	66.6 (9.1)	53-82
Educational level	11.9 (5.0)	3-18
Mini-Mental State Exam	21.1 (6.1)	11-29

* SD: standard deviation.

**Figure 1:**
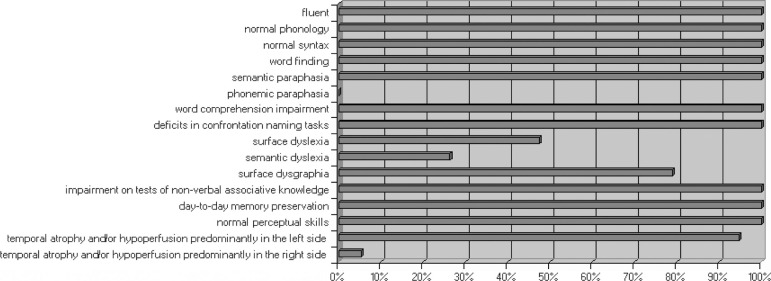
Frequency of main linguistic, cognitive and neuroimaging findings of nineteen patients with semantic dementia.

Figure 1 shows the frequency of main linguistic, cognitive and neuroimaging findings of 19 patients with semantic dementia. All the patients presented in spontaneous speech: speech fluency, preservation of syntactic and phonological language aspects, word-finding difficulty. Semantic paraphasias were observed in all patients, unlike the phonemic paraphasias which were not verified in any patients. Formal tasks revealed: word comprehension difficulty, low performance in visual confrontation naming tasks, impairment on tests of non-verbal semantic memory and preservation of the autobiographical memory and of visuospatial skills.

Regarding writing abilities, surface dysgraphia was observed in majority of the patients (78.9%). In relation to reading, surface dyslexia was verified in almost half of the patients (47.4%) and the semantic dyslexia in 33.3%.

Table 2 shows the patients’ performance in repetition, oral comprehension, naming and verbal fluency tasks. It can be observed that our case series had similar performance in word repetition, but the same was not observed in sentence repetition. The dissociation between performances comparing word comprehension (semantic) and the sentence comprehension (syntactic) is evident. All patients had better performance in sentence comprehension tasks than in semantic comprehension. Moreover, variability of intensity of semantic comprehension impairment was observed through minimum and maximum values: some patients presented intense difficulties and others, mild difficulties. In relation to syntactic comprehension, seven patients obtained maximum performance in the sentence comprehension task and 12 patients (63.2%) obtained performance of over 90% correct answers. Low performance of some patients on the sentence comprehension tasks was due to interference from semantic impairment. To exemplify, the patient that presented the lowest performance in the sentence comprehension task queried, during execution of the task, the meaning of words that composed the sentences of the test such as: “pushes”, “pulls”, “proceeds.” The marked semantic difficulty of this patient was obviously also verified in the word comprehension task. She obtained only 10% correct responses. In visual confrontation naming task, all patients presented low performance while in tasks of verbal fluency, the patients evoked low numbers of elements where difficulty was more intense in the category than in the letter fluency (FAS).

**Table 2:** Performance of semantic dementia patients on language tasks.

	Mean (SD*)	Minimum/maximal values	Median
Word repetition	99.7% (1.0)	96.7%-100.0%	100.0%
Sentence repetition	69.0% (23.2)	16.3%-100.0%	75.0%
Oral comprehension (words)	72.4% (20.8)	10.0%-95.5%	73.3%
Oral comprehension (sentences)	89.2% (17.0)	34.2%-100.0%	97.4%
Boston Naming Test (60)	10.4 (8.1)	1-30	9
Category fluency (animals)	4.4 (3.4)	0-10	4
Category fluency (utensils)	4.1 (4.7)	0-18	3
Letter fluency (FAS/3)	4.9 (4.1)	0-18.3	4.3

* SD: standard deviation.

**Figure 2:**
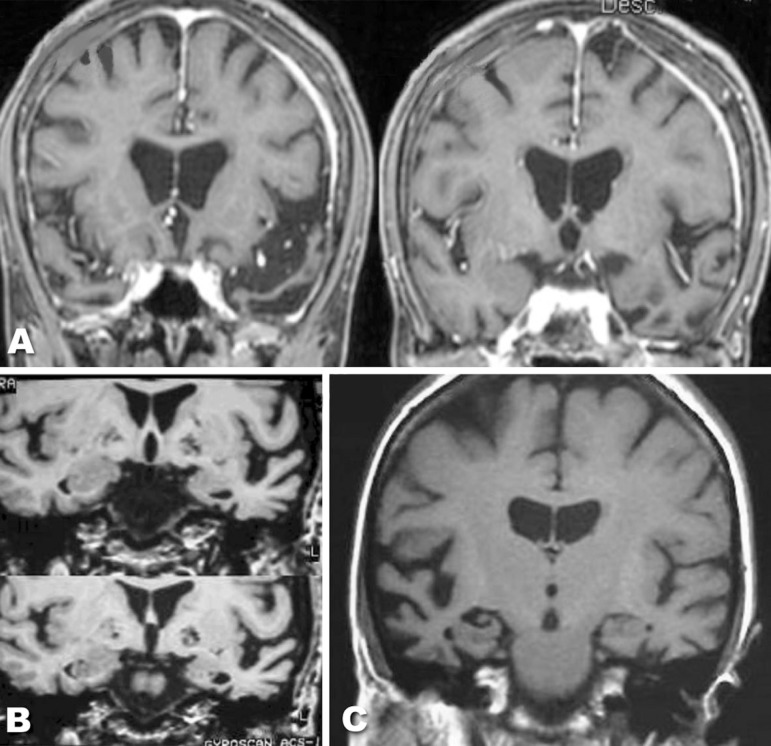
Examples of structural imaging (MRI) of three cases with semantic dementia. (A) and (B): bilateral anterior temporal lobe atrophy, which was more prominent on the left side; (C) bilateral temporal lobe atrophy, which was more prominent on the right side.

Regarding the neuroimaging assessment, involvement of temporal lobes (atrophy and/or hypoperfusion) was found in all patients (Figure 1). Most of the patients presented temporal lobe atrophy, which was more prominent on the left side, and only one case presented temporal lobe atrophy and hypoperfusion predominantly in the right hemisphere. Figure 2 shows MRI images of some cases. Hypoperfusion limited to the temporal lobes was found in most patients, however three cases presented extension of the hypoperfusion to the parietal lobe, and two cases to the frontal lobe.

Majority of the patients presented no behavior alterations. However, two cases presented disinhibition since the first evaluation. A further 2 patients out of 7 who were followed up, presented behavioral alterations with progression of the disease.

## Discussion

In our sample, in spite of the fact that most patients had disease onset in the presenile phase, the average age of onset was higher than other studies in the literature.^3,6,14,22^ The performance and variability found in scores on the MMSE^20,21^ can be attributed to the different degrees of semantic deficits and verbal production. The impact of the language disturbance should be considered, in the patients’ cognitive evaluation with PPA and SD, in interpreting the findings, because formal tests of neuropsychological batteries often depend on verbal comprehension of instructions, verbal responses or covert verbal reasoning.^1,2^ In PPA and SD cases, the evaluations of autobiographical memory and activities of daily living should be valued.

The linguistic and cognitive findings showed that the patients constituted a group with very homogeneous characteristics (Figure 1). This homogeneity can be explained taking into account that the primordial alteration that occurs in SD is semantic memory dissolution. This semantic deficit explains the anomia, low performance in word comprehension, verbal fluency tasks, both reading and written by lexico-semantic processes, and impairment in tests of non-verbal semantic memory. Nevertheless, akin to SD the impairment is limited to the semantic memory where patients have good performance in abilities not depending on semantic memory for instance, autobiographical memory, visuospatial skills and word repetition.

In surface dysgraphia and dyslexia, writing and the reading, respectively, are accomplished mainly by phoneme-grapheme conversion in the writing, and grapheme-phoneme conversion in the reading due to the semantic impairment. Consequently, patients with surface dysgraphia have difficulty in writing irregular words correctly in spite of preservation of writing regular words and non-words. The same occurs in patients with surface dyslexia that preserve the capacity to read regular words and non-words alongside the difficulty in reading irregular words. The regularization mistakes, that is, the application of the rules of conversion of the language, are pathognomonic symptoms of surface dysgraphia and dyslexia. As shown Figure 1, surface dysgraphia was not diagnosed in four of the 19 patients, however only one case did not present indications of isolated errors in the lexicon-semantic processing of the writing. This subject however presented a significant disturbance of reading and writing. Another SD case with similar intense disturbance of reading and writing as our patient has been previously described.^9^ The other three patients not diagnosed as surface dysgraphics, presented regularizations in the writing of irregular words and were capable of writing through conversion, but we ruled out surface dysgraphia diagnosis in these patients, because two of them had low education (three years) and the other patient was a foreigner and had not received formal education in the Portuguese language. Therefore, the regularization mistakes found in these patients may not necessarily have reflected a pathological process, but rather an educational process.

Regarding reading, surface dyslexia was verified in almost half of the patients (47.4%) and semantic dyslexia in 26.3% of patients. The frequent co-occurrence of surface dyslexia and semantic dementia has been raised and discussed by several researchers,^23-31^ and in 1992, Hodges et al.^9^ included the presence of surface dyslexia as one of the criteria for the diagnosis of semantic dementia. However, we propose that the semantic dyslexia can also be one of the manifestations of the semantic dementia, and their manifestations can be explained by three reading routes based on cognitive models.^32^ Semantic dyslexia is characterized by the reading possibility through the grapheme-phoneme conversion process and by the possibility of direct lexical reading without access to the semantic system. Thus, patients with semantic dyslexia read irregular words correctly, but they do not access the meaning of the word.

The reasoning mentioned previously behind the impact of the disturbance of the semantic memory in different linguistic abilities, among them, naming and word comprehension, that take place in SD was put forward by Warrington in 1975.^33^ From the concepts established by Tulving^34^ between the distinction among the long term memories in semantic and episodic memories, Warrington reported for the first time, three cases of degenerative disease with selective semantic memory impairment in conjunction with relative preservation of episodic memory. The patients seen by Warrington,^33^ who would now be classified as SD, presented difficulties in recognizing objects in the absence of a sensorial alteration. Moreover, the cases also presented difficulty in understanding the meaning of words and in naming objects and pictures. Other linguistic abilities were preserved as well as episodic memory and visuospatial ability.

This feature of SD - selective semantic memory impairment together with relative preservation of episodic memory - differentiates SD from Alzheimer’s disease that is characterized in the initial phase by episodic memory impairment. Therefore, it is necessary to be attentive to patient’ memory complaints during the anamnesis, investigating if their complaints and problems stem from semantic memory or episodic memory impairment.

Besides linguistic and cognitive homogeneous characteristics, our patients also presented a relatively similar atrophy pattern: all the patients presented temporal lobe involvement, most with predominant atrophy to the left side. One of our patients presented temporal atrophy predominantly in the right hemisphere. These findings mirror those described in the literature.^10,14,35-39^

The cognitive, linguistic and neuroimaging data of our case series corroborates other studies showing that SD seems to constitute a syndrome with well defined clinical characteristics associated to temporal lobe atrophy.^12,14,37,39^

Regarding the differentiation between fPPA and SD, it is important to bear in mind the views of several researchers studying this area. A consensus on classification of PPA has not yet been well established.^37-40^ Our patients can be classified as SD, and not as fPPA, since they presented language semantic disturbance and impairment on tests of non-verbal semantic memory. On the other hand, some researchers could argue that the impairment on the nonverbal semantic memory tests seen in most of our patients does not interfere in their activities of daily living and therefore, their difficulties mainly involve linguistic abilities and thus meet the diagnostic criteria of fPPA. According to our experience, fPPA cases with disturbance similar to semantic aphasia but without non-verbal semantic impairment can develop SD^19^ with progression of the disease. However, we disagreed with the view that every fPPA is an early SD.

The differential diagnosis between neurodegenerative diseases with prevalence of language (SD and PPA) and other neurodegenerative diseases such as Alzheimer’s disease is of great importance to the patient and their relatives. SD and PPA patients can be capable, after onset of symptoms, of maintaining many of their functional and even work activities.

## APPENDIX

### Illustrative case report

**Case SD2** – A 65-year-old right-handed, retired teacher, presented 3 year history of word finding difficulty together with impaired word comprehension and impaired people recognition (prosopoagnosia). The onset of the disease was slowly insidious with steady worsening. Day-to-day and personal autobiographic memories were unaffected. She continued taking care of her house without difficulty, went shopping in supermarkets and remained able to drive. She did not present depression signs. In the neurological exam, except for the alterations in semantic memory, no other abnormalities were seen. An MRI revealed bilateral temporal atrophy, more marked on the right, including hippocampal atrophy, most intense on the right side. A SPECT scan showed hypoperfusion confined to the right temporal lobe.

Neuropsychological testing revealed a decline in the Dementia Rating Scale – 116/144. Her score on the MMSE was 26/30. There was discrepancy between Verbal and Performance IQ in WAIS scores, which was more marked for verbal (102) than performance (106). Digit Span was 8 forward, 4 backward. Her day-to-day memory was unaffected. Constructional ability was preserved (copy of the Complex Rey-Osterrieth figure: 36/36).

SD2’s spontaneous speech was fluent and anomic with normal syntax, phonology and prosody. Comprehension of syntax was normal (Beta MT-86 protocol). However, oral and written comprehension of single words was impaired. Performance on word and non-word repetition tasks of the Beta MT-86 protocol was perfect while the result in the sentence repetition of Boston Diagnostic Aphasia Examination was close to normal. Her greatest difficulty was in the picture naming (9/60 correct answers in the Boston Naming Test). In this test, majority of the mistakes were due to inappropriate visual semantic recognition as in the following examples: [a] octopus: “Is it a fruit? I don’t know what it is? It isn’t an animal and it isn’t a plant either.” and [b] volcano: “A fire, but what kind of fire? What is being burned?” The second most frequent type of error was circumlocution: [a] hanger: “Where one puts the clothes ... how I forget the name...” and [b] racket: “Thing to play tennis, how can we say this?” As expected a disproportionate impairment of category rather than letter-based fluency was observed in the verbal fluency task. Among the category fluency tests, SD2 presented less difficulty in artifacts. Regarding reading and writing abilities, the patient presented surface dysgraphia and semantic dyslexia (she was able to read irregular and foreign words appropriately, but without comprehension). The difficulties in the nonverbal semantic memory were also evidenced in tasks of visual sorting, face recognition and visual semantic matching.
